# Invasive Mechanisms of One of the World’s Worst Alien Plant Species *Mimosa pigra* and Its Management

**DOI:** 10.3390/plants12101960

**Published:** 2023-05-11

**Authors:** Hisashi Kato-Noguchi

**Affiliations:** Department of Applied Biological Science, Faculty of Agriculture, Kagawa University, Miki 761-0795, Japan; kato.hisashi@kagawa-u.ac.jp

**Keywords:** allelopathy, biological control, monospecific stand, mutualism, natural enemy, phytochemical, rhizobium nodulation

## Abstract

*Mimosa pigra* is native to Tropical America, and it has naturalized in many other countries especially in Australia, Eastern and Southern Africa and South Asia. The species is listed in the top 100 of the world’s worst invasive alien species and is listed as Least Concern in the IUCN Red List of Threatened Species. *M. pigra* forms very large monospecific stands in a wet–dry tropical climate with conditions such as floodplains, riverbanks, grasslands, forests and agricultural fields. The stands expand quickly and threaten the native flora and fauna in the invasive ranges. Possible mechanisms of the invasion of the species have been investigated and accumulated in the literature. The characteristics of the life history such as the high reproduction and high growth rate, vigorous mutualism with rhizobia and arbuscular mycorrhizal fungi, very few natural enemies, and allelopathy, and certain secondary metabolites may contribute to the invasiveness and naturalization of *M. pigra*. Herbicide application, such as aerial spraying, foliar, cut-stump and soil treatments, is the primary control methods of *M. pigra*. The investigation of the natural enemies of *M. pigra* has been conducted in its native ranges since 1979, and biological control agents have been selected based on host specificity, rearing and availability. Mechanical control practices, such as hand weeding, bulldozing, chaining and fire, were also effective. However, the species often regrow from the remaining plant parts. Integration of multiple weed control practices may be more effective than any single practice. This is the first review article focusing on the invasive mechanism of *M. pigra*.

## 1. Introduction

**Figure 1:**
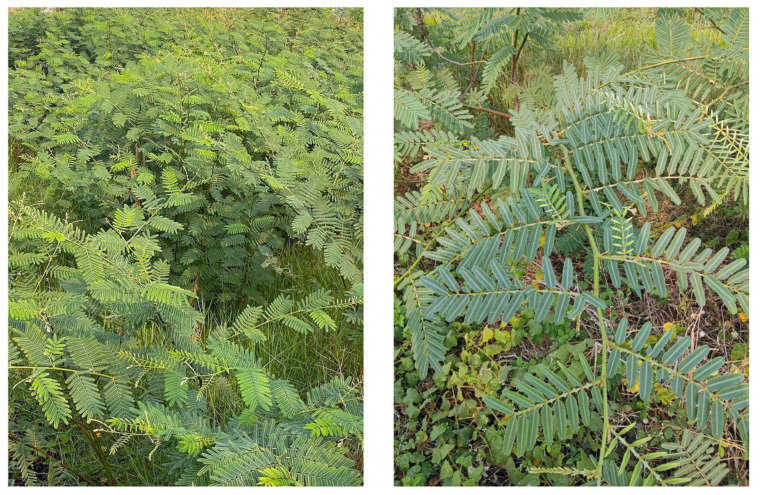
*Mimosa pigra* stand and leaves.

*Mimosa pigra* L. (Syn. *Mimosa pellita* Kunch ex Willd.) belongs to the Fabaceae family, is known as a giant sensitive plant, and is listed in the top 100 of the world’s worst invasive species [1,2] and is listed as Least Concern in the IUCN Red List of the Threatened Species [3]. *M. pigra* grows well under wet–dry tropical climate conditions [1,2,4]. The species is shrubby, highly branched up to 6 m in height and forms dense monospecific stands with an average density of one plant per m^2^ on riverbanks, floodplains, swamp forests, coastland, canals, agricultural fields and roadsides [4,5]. It has alternate and bipinnate leaves (ca. 25 cm long) with 10–16 pairs of opposite pinnae (ca. 5 cm long). The pinnae contain narrow lanceolate leaflets, and the leaflets close together in the evening and when touched and injured (Figure 1). Its stems contain randomly scattered prickles (ca. 1 cm long) [4,5]. 

### 1.1. Invasiveness of the Species

*M. pigra* is native to Tropical America, and it has been introduced into some other countries as an ornamental plant, green manure, cover crop and seed contaminant [1,2,6,7]. The species has spread and naturalized in many countries especially in Australia, Eastern and Southern Africa and South Asia [1,3,8]. *M. pigra* was thought to be introduced to the Darwin Botanical Gardens in the Northern Territory of Australia as an ornamental plant before the 1980s. The species reached the open floodplains in the 1970s and spread and formed the monospecific stands that eventually covered over 80,000 ha of the wetlands in the Northern Territory of Australia. Most of Adelaide River floodplains were occupied by its monospecific stands by 1981 [6,9,10]. The species was confirmed to be present in the coastal floodplain of the Northern Territory, and it expanded from Western Australia into Queensland [11].

The first observation of *M. pigra* in Africa was made in Nigeria in 1822, and its infestations increased quickly. The rapid expansion of *M. pigra* was recorded in the Kafue floodplain of Zambia. The species occupied only 2 ha in the floodplain by 1980, and then spread quickly, covering 100 ha by 1986 and 2500 ha by 2003, becoming the most dominant plant species in the floodplain [12,13,14]. The species has already naturalized in all Eastern and Southern Africa countries except for Lesotho and Swaziland [1,2,15].

*M. pigra* was identified in the Mahawei River Bank in Sri Lanka in the early 1980s, and occupied the riverbank along one km and the adjacent lowland area by 1996. The subsequent survey showed that the species formed a dense thickness along 30–35 km of the riverbank of the Mahawei River and spread into 46 locations in three provinces including other riverbanks, abandoned paddy fields, roadsides and home gardens [16]. The species was introduced to Thailand in 1947 as a cover crop and as green manure from Indonesia, and it spread extensively and covered large areas of riverbanks and floodplains throughout the country [17]. *M. pigra* has infested the Mekong River Basin since the 1970s and has spread over 30% floodplain areas of the Mekong River [18]. The genetic variation of the *M. pigra* population indicates multiple introductions of the species into Thailand [19]. The species is also spreading in other South Asian countries [1,2,20,21].

The invasion of *M. pigra* has large negative impacts on the natural ecosystems and agricultural and fishing industries [1,15,22,23]. *M. pigra* thickets prevent the regeneration process of other plant species including indigenous plant species within the understory [24]. The species was observed to replace previous dominant grass species such as *Echinochloa stagnina* (Retz.) P. Beauv. and *Oryza longistaminata* A. Chev. and Roehr. in the Kafue floodplain of Zambia [13]. Dense monospecific stands of *M. pigra* prevent incident light penetration into the soil surface [25], which may be one of the causes for fewer numbers of indigenous plant species in its infestations [4,22]. 

### 1.2. Impact of the Species

*M. pigra* thickets disturb wildlife movements and their water availability from the water front and decrease available grazing areas for herbivores and related carnivores including birds and lizards [4,15,22,25]. The population of large water birds in the *M. pigra* infestation areas of the Bueng Boraphet wetland in Thailand was reduced by 27% during 2003 to 2014 with the highest decline of 56% in ducks [26]. The bird population was 20,000 in the native area in the Kafue floodplains of Zambia, but it was only 314 in the *M. pigra* infested areas, which was a 50% reduction in bird species [14]. *M. pigra* thickets also reduced water flow and increased silt levels, resulting in the conversion of the floodplains into scrubland [15], which may be one of the causes for the reduction of the biodiversity and population. 

*M. pigra* also infested agricultural fields, grasslands and forests [1,4,15]. *M. pigra* thickets reduced the farmland and blocked irrigation systems in rice paddy and other crop fields, reducing crop production [15,27,28]. Average famers spend 11 days per ha annually to clear the species in their farmlands in the Mekong River floodplains [18]. The species suppressed the growth of oil palm, resulting in decreased palm oil production [29]. *M. pigra* thickets reduced the grazing areas for livestock and blocked livestock’s access to water sources [4,15]. The fishing production in the river near the floodplains was also decreased by the invasion [15]. Therefore, the invasion of *M. pigra* puts natural ecosystems at high risk by reducing the native biodiversity and population. It also causes reductions in agricultural and fishing production [14,15]. There have been some recent review articles on the impact of *M. pigra* invasion and its management [15,23]. The species thrives under a wet–dry tropical climate condition [1,2,4], which indicates that global warming may increase the threat of the invasion of the species into additional non-native areas. However, no review article on the invasive mechanism of *M. pigra* is available. The objective of this review is to discuss possible invasive mechanisms of *M. pigra* and its management practices. The paper provides an overview of the literature, describing the invasive mechanisms of the species and management practices. 

## 2. Invasive Mechanism 

### 2.1. Reproduction and Growth

**Figure 2:**
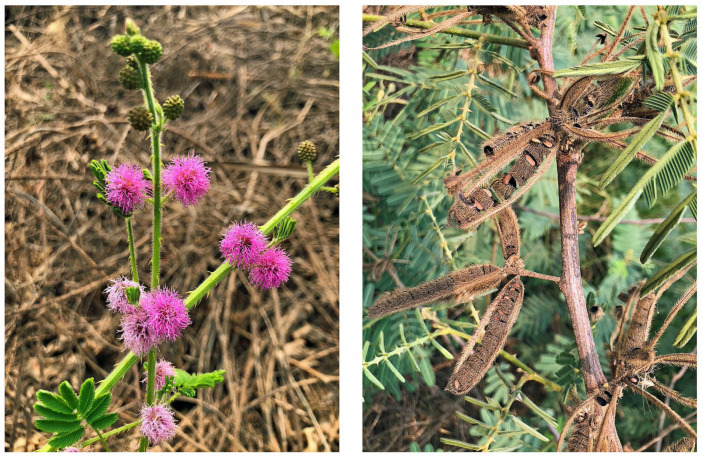
*Mimosa pigra* flowers and pods.

*M. pigra* is a fast-growing species, and it is capable of reaching reproductive maturity within 6–8 months [4,5,16,30,31]. Round flower heads (1–2 cm in diameter; mauve or pink) arise from actively growing young shoots, which contain approximately 100 flowers. Each flower head generates 1–30 seed pods. Pods are 3–8 cm in length and are covered with dense stiff hair (Figure 2). Each pod contains oblong-shaped 8–20 seeds (4–5 mm long, 2 mm wide) [4,5,31]. The species flowers throughout the year in Sri Lanka and Queensland, Australia, and during spring to autumn in the Northern Territory of Australia, which may be dependent on climate conditions [5,16,30]. Flowers are pollinated by mostly self-fertilization and sometimes by bee or wind [4]. The seeds take about five to nine weeks for the maturation after the flower-bud formation [5,31] (Figure 2).

Annual seed production was estimated to be up to 220,000 seeds per plant [31,32], and 9000–12,000 per m^2^ [33]. Top soil under the canopy contained 2000 to 45,000 seeds per m^2^ [12,16,33]. The wind dispersion of the seeds occurs a relatively short distance from the plants. The long-distance dispersion of the seeds occurs through the adhesion of the pod’s stiff hair onto animals and agricultural vehicles and through the floating of the pods on water streams and flooded waters [12]. 

The seeds germinate when they are first wetted, and the rate of the germination is 75–94% [34]. The half-life of viable seeds in seed banks in field conditions is 9–99 weeks, which is dependent on the soil types and conditions [33,35]. The seed coats are very hard and impermeable and some of seeds have remained dormant in the soil for up to 26 years [31,36]. Ten years after the complete clearance of 250 ha of a *M. pigra* stand, its 109 seedlings per m^2^ still remained to emerge from the seed banks [36]. Sand scarification of the seeds increased the germination [16], indicating that the movements of the seeds by water stream and flooding may stimulate the germination.

The species grows at a rate of 1.1 cm in height per day during the first 90 days after germination and grows ca. 2.5 cm and ca. 7.5 cm in the stem diameter in the first year and the second year, respectively [4,5,37,38]. The species forms impenetrable dense monospecific stands (3–6 m in high) and the stands expand 76 m per year in wetlands [12]. It was recorded that the infested areas of the active stands doubled in 1 year and on average every 6 years in the Northern Territory of Australia [12,31]. The coverage of *M. pigra* in the monospecific stands was 96.3% and the biomass was estimated to be 35–45 tons dry weight per ha in the Adelaide River floodplain and the Finniss River catchment in Australia [39,40]. 

The species also regenerates from the remaining trumps after clearance of the above-ground parts of the trees [34]. Substantial numbers of the plants regrow from the base of stems after fire burning and the fire stimulates its germination in the seed banks [41]. The regrowth from the young stubble can reach 2.5 m in height and can cover 6.3 m^2^ within 12 weeks [38]. 

The characteristics of life history such as the high reproduction and high growth rate are important for the invasiveness and naturalization of invasive plants [42,43,44,45]. The observations described in this section suggest that *M. pigra* has the ability of rapid growth through its vegetative phase to flowering, self-compatibility, high seed output, high rate of germination, great longevity of seeds and regrowth from the stubbles (Table 1). These characteristics may contribute the invasion and naturalization of the species in invasive ranges.

### 2.2. Adaptivity and Plasticity

The species is found in tropical regions where annual rainfall level is between 750 mm and 2200 mm. It can grow around water bodies even when the annual rainfall is less than 750 mm [5]. *M. pigra* grows well in soil ranging from black cracking clays, sandy clays and siliceous river sand, although the species can grow in any type of soil [4,32]. It is found at an altitude of ca. 500 m above sea levels [37]. *M. pigra* has shown phenotypic plasticity in response to abiotic and biotic stress conditions such as available water level and intraspecific competition [46]. The genetic variation and structure of the *M. pigra* population in Thailand is high [19]. The characteristics of phenotypic plasticity of the plants are important for the naturalization of invasive plants into non-native ranges [42,43,44,45,46]. However, information is limited to discuss the phenotypic plasticity of *M. pigra* in different environmental conditions.

### 2.3. Natural Enemy

Long-term investigations from 1979 in the native ranges of *M. pigra* such as in Central and South America and in Mexico have shown that over 400 phytophagous insects, consisting of 61 families in 5 orders, are the natural enemies of *M. pigra*. The largest family is Coleoptera (59%), followed by Hemiptera (23%) and Lepidoptera (17%) [47,48]. Among them, for example, a stem-boring moth *Carmenta mimosa* Eichilin and Passoa (Lepidoptera) caused a 90% reduction in the seed production of *M. pigra*, and a weevil *Coelocephalapion pigrae* Kissinger (Coleoptera) rapidly colonized the *M. pigra* stands and fed on their leaves [49,50].

Pathogenic fungi: *Mycosphaerella mimosae-pigrae* H. C. Evans, G. Carrión and Ruiz-Belin; *Sphaerulina mimosae-pigrae* H. C. Evans and G. Carrión; *Diabole cubensis* Arthur and J. R. Johnst.; and *Microstroma ruizii-belinii* H. C. Evans, G. Carrión and Ruiz-Belin were found to infect *M. pigra* along the Pacific Coast of Mexico, and *Sphaerulina mimosae-pigrae* and *Diabole cubensis* occurred along the Caribbean Coast [51,52]. *Phloeospora momosa-pigrae* H. C. Evans and G. Carrión and *Diabole cubensis* selectively infected *M. pigra* in Mexico [31,51]. Some of those natural enemies were selected as the biological control agents for *M. pigra,* which were described in Section 3.3.

The interactions of the invasive plants with natural enemies are very critical for the naturalization of the invasive plants [42,43,44,45,46]. A great number of herbivore insects and fungal pathogens have been identified in *M. pigra* stands in the native ranges described above. However, very few natural enemies were found in Australia [53]. Having few natural enemies may contribute to the superior growth rate and naturalization of *M. pigra* in invasive ranges (Table 1). 

### 2.4. Mutualism

Plant species belongs to *Mimosa* genus nodulate generally with the member of the *Betaproteobacteria* (β-rhizobia or β-proteobacteria), which includes the genera of *Cupriavidus, Burkholderia*, *Paraburkholderia* and *Trinickia* [54,55]. The species of *Burkholderia* was the main symbiosis rhizobia for *M. pigra*, followed by the species of *Cupriavidus* in South and Central America and in Taiwan [56,57,58]. Among 191 rhizobia isolated from the root nodules from three separated populations of *M. pigra* in Taiwan, 96% and 4% of rhizobia were members of *Burkholderia* and *Cupriavidus*, respectively [59]. 

Rhizobium nodulation enhances the host plant performance through the nitrogen and ammonium supply to host plants [60,61]. The nitrogen-fixing ability of *Burkholderia* species nodulated with *M. pigra* was also much greater than that of *Cupriavidus* species [59]. *M. pigra* nodulated vigorously even under flooded condition and fixed substantial quantities of nitrogen [62,63,64]. 

Rhizobium species, *Burkholderia mimosarum* sp. nov. was isolated from the root nodules of *M. pigra* population in Taiwan and Venezuela. However, the strains of *Burkholderia mimosarum* sp. nov. from Taiwan (invasive range of *M. pigra*) differed from the strain from Venezuela (native range of *M. pigra*) [57,59,65]. The strain LMG 23256^T^ of *Burkholderia mimosarum* sp. nov., which was isolated from the root nodules of the *M. pigra* population in Taiwan, was highly effective for nitrogen fixing than the strains from Venezuela [66]. The Taiwan strains showed fast growing and fast colony-forming ability [67,68] and outcompeted other rhizobium species for nodulation with *M. pigra* under flooded conditions [58].

Ninety rhizobia isolated from the root nodules of *M. pigra* in an Australia population (i.e., an invasive range) were characterized as *Burkholderia* spp., which are also the main rhizobia in Tropical America (i.e., native ranges) [56,57,58]. The strains of *Burkholderia* in Australia showed divergent lineages, and all of them did not have a close relationship to the *Burkholderia* strains in the native ranges. Inoculation of *M. pigra* with the Australian *Burkholderia* strains showed equal or higher nodule nitrogenase activity than that of with the Tropical American *Burkholderia* strains, which resulted in its high plant growth rate. Therefore, the *M. pigra* population in Australia acquired more effective nitrogen-fixing symbionts compared to the *M. pigra* population in the native ranges [69].

A high level of arbuscular mycorrhizal fugus colonization was found in the flooded roots of *M. pigra* in wetlands [70,71]. The dominant mycorrhizal fungi in the *M. pigra* roots are the members of the *Rhizophagus* and *Glomus* genera, which belong to the *Glomerales* order and are considered to be generalist mycorrhizal fungi [72]. Arbuscular mycorrhizal fungi enhance their host plant performance through increasing water and nutrient acquisition, photosynthetic activity, and defense functions against the pathogen attacks and stress conditions [73,74,75]. Arbuscular mycorrhizal fungi also improve the host plant performance even in wetland conditions [76]. 

Those observations suggest that *M. pigra* associates actively with rhizobia and arbuscular mycorrhizal fungi even under flooded conditions. *M. pigra* in the invasive ranges may colonize with rhizobia, which possess high nitrogen-fixing activity compared to that of its native ranges (Table 1). The mutualism with rhizobia with high nitrogen-fixing activity in the invasive ranges may contribute to the invasiveness of the species.

### 2.5. Allelopathy

Many secondary metabolites in the invasive plants exhibit the function of allelopathy [77,78,79,80]. Allelopathy is the interaction between donor plants and their neighboring plants through certain secondary metabolites that are defined as allelochemicals [81,82,83,84]. The allelochemicals are released into the vicinity of the donor plants either by volatilization, rainfall leachates, root exudation and decomposition processes of donor plant residues, and they suppress the germination, growth and establishment of neighboring plants, as well as exhibiting mutualism with rhizobia and arbuscular mycorrhizal fungi [85,86,87,88,89,90,91]. Since allelochemicals are synthesized and stored in certain plant tissues until releasing into the vicinity of donor plants [81,82,83,84], several researchers determined the allelopathic activity in the residues of the leaves and extracts from different plant parts of *M. pigra*.

The leaf powder of *M. pigra* was mixed with soil and then the seeds of *Ruellia tuberosa* L. were sown into the mixture. The mixture suppressed the germination and growth of *Ruellia tuberosa* [92]. *Ruellia tuberosa* is also invasive species from Central and South America [93]. Aqueous extracts of *M. pigra* leaf powder inhibited the germination and growth of *Vigna radiata* (L.) R. Wilczek in an extract concentration-dependent manner [94]. The leaves and stems of *M. pigra* were soaked in boiling water for 10 min and the obtained solutions inhibited the root growth of *Allium cepa* L. and disturbed the cell division of its meristematic root cells, such as reducing the mitotic index and increasing chromosomal aberrations [95]. Methanol leaf extracts of *M. pigra* suppressed the root and shoot growth of *Ruellia tuberosa, Echinochloa crus-galli* (L.) P. Beauv. and *Lactuca sativa* L. The extracts showed the reduction of cell viability of their roots and also disturbed the mitosis of their root cells in a concentration-dependent manner. The extracts also increased lipid peroxidation in their roots and shoots [96,97]. 

Those observations suggest that the leaf residues and leaf and stem extracts of *M. pigra* exhibit allelopathic activity that influences the germination and growth of some plant species, as well as probably contains water and methanol extractable allelochemicals. Some of these allelochemicals would be liberated into the soil during their decomposition processes of the residues. Total annual litterfall of *M. pigra* was estimated to be 758 g m^2^ [98], and such a litterfall may be one of the sources of allelochemicals of the species. Allelochemicals of the invasive plant species suppressed the regeneration process of the native plant species in their invasive ranges [77,78,79,80,81,82,83,84,85,86,87,88,89,90,91]. Allelochemicals of *M. pigra* may also suppress the regeneration process of the native plant species through the inhibition of their germination and growth. Total concentrations of flavonoids, tannins and saponins were estimated in *M. pigra* leaves [97]. However, there has been no information available on the isolation and identification of the allelochemicals from *M. pigra*. 

Mimosine (synonym; leucenol) was first isolated from *Mimosa pudica* L. [99] and found in some other species of the *Mimosa* and *Leucaena* genera [100,101,102]. Mimosine has shown a wide range of biological properties such as allelopathic, anti-tumor, apoptotic, anti-inflammation, anti-viral, and cell cycle blocking activity [103]. However, mimosine has not yet been identified in *M. pigra.*

### 2.6. Secondary Metabolites

**Figure 3:**
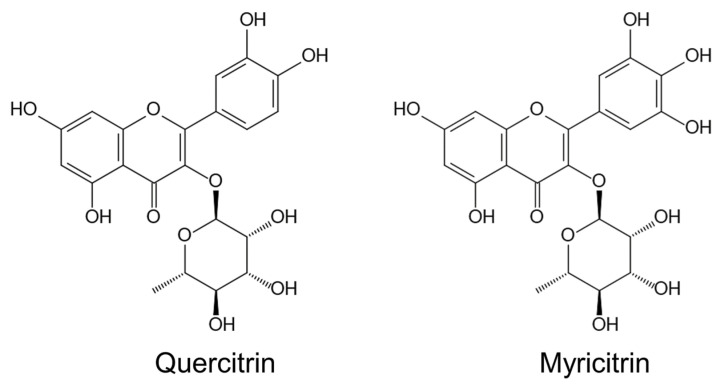
Possible allelochemicals of *Mimosa pigra*.

Pharmacological investigations showed that *M. pigra* contains several secondary metabolites, which have pharmacological activity such as analgesic, antipyretic, anti-inflammatory, anti-diabetic, anticancer and antioxidant activity [104,105,106]. The methanol extracts of *M. pigra* leaves showed antioxidant and anti-inflammatory actions in the Wister rat, and quercitrin (quercetin 3-*O*-rhamnoside) and myricitrin (myricetin 3-*O*-rhamnoside) were isolated from the extracts [104] (Figure 3). The methanol extracts of *M. pigra* leaves also showed anti-dermatophyte activity, and astragalin, luteolin and quercitrin were isolated from the extracts [105]. 

Several flavonol glycosides, quercetin (2″-*O*-galloyl)-3-*O*-α-L-rhamnopyranoside, quercetin-3-*O*-α-L-rhamnopyranoside, quercetin-3-*O*-α-L-arabinopyranoside, myricetin (2″-*O*-galloyl)-3-*O*-α-L-rhamnopyranoside and myricetin-3-*O*-α-L-rhamnopyranoside [107], and quercetin-3-*O*-α-L-rhamnopyranoside, quercetin-3-*O*-β-D-galactopyranoside, quercetin-3-*O*-α-L-arabinopyranoside, myricetin-3-*O*-α-L-rhamnopyranoside, kaempferol-3-*O*-α-L-rhamnopyranoside and kaempferol-3-*O*-α-L-rhamnopyranosyl-(1→2)-β-D-glucopyranoside, and a flavonoid; 3′,4′,5,7-tetrahydroxyflavone [108], were isolated and identified in the leaf extracts. Terpenoid saponin: machaerinic acid was isolated from stems of *M. pigra* [109]. A furanochromone, 6,8-dihydroxy-2-methyl-9*H*-furo(3,2-*b*)choromen-9-one, was isolated and identified in the leaf extracts of *M. pigra* [110]. Pharmacological active compounds identified in the plant species of *Mimosa* genus were also reviewed by Rizwan et al. [106].

**Table 1:** Possible mechanisms for the *Mimosa pigra* invasion.

Characteristic	Reference
Rapid growth through the vegetative phase to flowering	[4,5,16,30,31]
Self-compatible	[4]
High seed output	[12,16,31,32,33,34]
High germination rate	[5,16,34]
Longevity of seeds	[31,36]
Regeneration from remaining plant parts	[34,41]
Vigorous mutualism with rhizobia and arbuscular mycorrhizal fungi	[62,63,64,70,71]
Colonizing with rhizobia with high nitrogen-fixing activity in the invasive ranges	[58,66,67,68,69]
Very few natural enemies in the invasive ranges	[53]
Allelopathy	[92,93,94,95,96,97]
Secondary metabolites	[104,105,106,107,108,109,110]

Many flavonoids have shown anti-herbivore, anti-fungal and anti-bacterial activity [111,112,113]. In addition, quercitrin and myricitrin have also been isolated from *Ludwigia hexapetala* Hook. (i.e., water primrose), and they displayed allelopathic activity [114]. *Ludwigia hexapetala* is native to Central and South America, it is a noxious invasive species in Western Europe and the United States and it grows well in swampy lands such as the margins of lakes and streams [115]. Quercitrin was reported to work as an allelopathic agent for the other territorial plant kiwifruit (*Actinidia deliciosa* (A. Chev.) C. F. Liang et A. R. Ferguson), and it has inhibited the growth of several other plant species [116,117]. 

Although most of the identified compounds in *M. pigra* have not yet been related to the invasiveness of the plant species, some of them may be involved in allelopathy and defense functions against herbivores and pathogenic fungi. Therefore, these compounds may contribute to the invasiveness and naturalization of *M. pigra* in the invasive ranges.

## 3. Management 

The efforts of the prevention of the *M. pigra* invasion are generally focused on the major infestation sites, although small satellite populations often grow and disturb the indigenous ecosystems more quickly than the largest population [118]. Mechanical, chemical and biological approaches were made to control the major and satellite *M. pigra* populations [23,40,119].

### 3.1. Mechanical Control and Fire

Hand weeding and cutting can apply for the incipient outbreaks and the isolated infestations of *M. pigra.* One-third of the population of *M. pigra* infestations was eradicated within one year, with sustained control thought to be necessary for at least 7 years to prevent regeneration from seed banks [40]. The regrowth from the stubble of *M. pigra* also occurs quickly [4,38,40]. Bulldozing, chaining and ploughing can be used for relatively large infestations [11,119]. *M. pigra* is difficult to burn by moderate fire, with substantial numbers of plants regrowing from the stem base after burning [41,120]. Burning has also enhanced its seed germination [41]. Burning efficiency depends on several factors such as the timing of the fire treatments, intensity of the fire and the target weed species [121,122]. However, these treatments may affect the native flora and fauna, so the influence of the treatments on the environments needs to be considered.

### 3.2. Chemical Control

Herbicide application, such as aerial spraying and foliar, cut-stump and soil treatments, is the primary control method of *M. pigra*. Metsulfuron-methyl, dicamba, fluroxypyr, hexazinone, tebuthiuron and glyphosate are the principal chemicals [23,123,124]. Metsulfuron-methyl (group 2, ALS inhibitor) is the most effective chemical for the juvenile plants by using aerial spraying [124]. Dicamba (group 4, auxin mimic) is recommended for actively growing plants by using aerial spraying and foliar treatments [124]. Fluroxypyr (group 4, Auxin mimic) is recommended for actively growing plants by using foliar treatment and cut-stump treatment mixed with diesel [40,124]. Hexazinone (group 5, PSII inhibitor-Serine 264 binder) is recommended for seedling and adult plants by using aerial spraying, but it is not recommended for continuous use in large areas as a non-selective residual herbicide [124]. Tebuthiuron (group 5, PSII inhibitor-Serine 264 binder) is recommended for actively growing plants before seed maturation by using aerial spraying and soil treatments [125]. Glyphosate (Group 9, inhibition of enolpyruvyl shikimate phosphate synthase) is recommended for the all-growing stage of the plants by using cut-stump treatment mixed with water [124]. These investigations suggest that several herbicides are available to control *M. pigra*, and the timing and manner of the treatments may be crucial to the herbicide application. In addition, there is a recent review article describing the chemical control of the species [23].

### 3.3. Biological Control

From 1983 to 2005, 12 insects (eight Coleoptera and four Lepidoptera) and two pathogenic fungi have been released in Australia. Among them, eight insects (five Coleoptera and three Lepidoptera) and one pathogenic fungus have been established [48,49,126,127] (Table 2). The stem-boring moth *Carmenta mimosa* Eichilin and Passoa is the most successive agent [50,126,128]. *Carmenta mimosa* was released in 1989 and found in most of the *M. pigra* infestations in 2004. High density of *Carmenta mimosa* larvae caused the reduction in the plant vigor with dead and broken branches, 90% seed rain and seed banks [50,128]. The abundance of the *Carmenta mimosa* population was negatively correlated with the population of *M. pigra* [50,126]. Another moth species *Neurostrota gunniella* Busck was also released in 1989, where it spread rapidly over the most of the *M. pigra* infestations [126,129]. Its larvae feed on the pinnules of the leaves and stems of *M. pigra* [130]. The population of the species was relatively stable and the most stems of *M. pigra* on the outside of stands were affected by the moth. The feeding of the larvae caused a reduction in the seedling growth and seed rain [131,132]. The seed rain from *M. pigra* was negatively correlated with the population of the larvae, and the larvae feeding decreased seedling growth by 30% [131]. The moth species *Macaria pallidata* Warren was released in 2002 and distributed among most of the *M. pigra* infestations. The larvae feed on the leaves of *M. pigra.* However, the rate of its parasitism was low [133].

The weevil species *Coelocephalapion pigrae* Kissinger was released in 1994 and was widespread in 2004 [126]. Its larvae feed on the flowers and leaves of *M. pigra,* and the population was relatively stable [134]. Another weevil species *Chalcodermus serripes* Fahreaus was released in 1996 and was once thought to have disappeared. However, the weevil was later found in the survey sites of the Northern Territory of Australia [127]. The larvae feed on the unmatured seed and leaves of *M. pigra* [126]. The bean weevil species *Acanthoscelides puniceus* Johnson was released in 1983. It was distributed widely in 1997 and found in 24% of the *M. pigra* infestations in 2004 [126]. Its larvae feed on the seeds of *M. pigra.* However, its impact of seed destruction was not high [129]. 

The leaf beetle species *Chlamisus mimosae* Karren was released in 1985 and was found in 8% of the *M. pigra* infestations in 2004. The larvae feed on the stems and leaves of *M. pigra.* However, the number of the larvae was low in each infestation site [126]. Another leaf beetle species, *Malacorhinus irregularis* Jacoby, was released in 2000. Its larvae feed on the leaves, roots and nodules of *M. pigra* [135]. However, the distribution of the species was limited [126]. Pathogenic fungus *Diabole cubensis* was released in 1996. It was thought to fail the establishment, but it was observed at several survey sites in 2011 and 2012 [136]. In addition to this achievement in Australia, two bean weevil species *Acanthoscelides quadridentatus* Schaeffer and *Acanthoscelides puniceus* Johnson were released in 1980–1981 and established in Thailand. These species feed on the seeds [137]. 

These observations suggest that *Carmenta mimosa, Neurostrota gunniella* and *Coelocephalapion pigrae* are widespread and abundant among the *M. pigra* infestations. These species are capable of feeding on the leaves and stems, which are available throughout the year. *Carmenta mimosa* showed the largest impact, and its population continued to increase [138]. *Carmenta mimosa* may contribute to shrinking the *M. pigra* stands. Biological control of invasive weeds is considered to be among the most environmentally friendly and cost efficiency practices to manage large scale weed infestations [139]. However, biological control alone did not provide adequate control output. Therefore, a combination with additional management options is necessary [119].

**Table 2:** Established biological control agents in Australia.

Species	Order	Group	Year of the First Release	Attacked Plant Part	Reference
*Carmenta mimosa*	Lepidoptera	Moth	1989	Stem	[50,126,128]
*Neurostrota gunniella*	Lepidoptera	Moth	1989	Stem, pinnules, seedling	[52,126,129,130,131,132]
*Macaria pallidata*	Lepidoptera	Moth	2002	Leaf	[133]
*Coelocephalapion pigrae*	Coleoptera	Weevil	1994	Flower, leaf	[126,134]
*Chalcodermus serripes*	Coleoptera	Weevil	1996	Seed, leaf	[126,137]
*Acanthoscelides puniceus*	Coleoptera	Bean weevil	1983	Seed	[126,129]
*Chlamisus mimosae*	Coleoptera	Leaf beetle	1985	Stem, leaf	[126]
*Malacorhinus irregularis*	Coleoptera	Leaf beetle	2000	Leaf, root, nodule	[126,135]
*Diabole cubensis*	Pucciniales	Fungus	1996	Leaf	[136]

### 3.4. Integrated Control

Single and repeated treatments of herbicides, fire and crushing by bulldozer with biological control agents were applied to a large-scale area (128 ha) of a *M. pigra* infestation. Any single treatment was not effective to control *M. pigra,* and the combination of these treatments was more effective to clear thickets of *M. pigra* and promote the establishment of competing native vegetation. The population of the biological agents, *Carmenta mimosa* and *Neurostrota gunniella*, on the surviving *M. pigra* was either unchanged or increased even after herbicide, fire and bulldozing treatments [50]. Integration of multiple weed control practices may be more effective than any single practice and it may produce a synergistic effect for controlling infestations [119,140]. The integration may also reduce the opportunity to develop herbicide resistant weed species [121]. The timing of the herbicides, fire and mechanical treatments affects the weed control implementation, as well as minimizes adverse effects of the biological control agents [141,142]. A review article focuses on integrated management for *M. pigra* [119]. The appropriate treatments methods and timing may vary based on the target weeds and scale of the infestations. 

## 4. Conclusions

*M. pigra* is highly invasive and has naturalized in wet–dry tropical climate conditions such as floodplains, riverbanks, coastland, canals, agricultural fields and roadsides. Very large monospecific stands of *M. pigra* are often observed in several countries in Australia, Eastern and Southern Africa and South Asia. The species showed rapid growth through its vegetative phase to flowering and a high reproduction rate and longevity of seeds. The species also associates vigorously with rhizobia and arbuscular mycorrhizal fungi even under flooded conditions, and it colonizes with rhizobia, which possesses higher nitrogen-fixing activity in its invasive ranges than in its native ranges. A great number of natural enemies of *M. pigra* were found in its native ranges, but very few natural enemies were found in its invasive ranges. *M. pigra* is allelopathic and contains possible allelochemicals such as quercitrin and myricitrin. Allelochemicals of the species may suppress the regeneration process of the native plant species through the inhibition of their germination and growth. *M. pigra* also contains several other flavonoids and its derivatives, some of which may have anti-herbivore, anti-fungal and anti-bacteria activity. The characteristics of its life history, such as high reproduction and high growth rate, vigorous mutualism with rhizobia and arbuscular mycorrhizal fungi, very few natural enemies, and allelopathy, and certain secondary metabolites, may contribute to the invasiveness and naturalization of *M. pigra*. However, information for the phenotypic plasticity of *M. pigra* is limited.

Among the natural enemies of *M. pigra* in its native ranges, some species were selected and released into the invasive ranges. The stem-boring moth *Carmenta mimosa* has been one of the most successive biological control agents. Metsulfuron-methyl, dicamba, fluroxypyr, hexazinone, tebuthiuron and glyphosate are the principal chemicals for chemical control. Mechanical control practices and fire are also effective management options. However, the species often regrows from the remaining plant parts such as the stubbles. Integration of multiple weed control practice may be more effective than any single practice, and such an approach may produce a synergistic effect for controlling *M. pigra*. The selection of weed control options for the integration and timing of these treatments is critical.

## References

[1] Global Invasive Species Database. Species Profile: *Mimosa pigra*. http://www.iucngisd.org/gisd/speciesname/Mimosa+pigra.

[2] Invasive Species Compendium, *Mimosa pigra*. https://www.cabidigitallibrary.org/doi/10.1079/cabicompendium.34199.

[3] IUCN Red List of Threatened Species, *Mimosa pigra*. https://www.iucnredlist.org/species/49486238/49486516.

[4] Beilfuss R. (2007). Adaptive Management of the Invasive Shrub Mimosa pigra at Gorongosa National Park.

[5] Lonsdale W.M., Miller I.L., Forno I.W. (1989). The biology of Australian weeds. 20. Mimosa pigra. Plant Prod. Q..

[6] Miller I.L., Lonsdale W.M. (1987). Early records of *Mimosa pigra* in the Northern Territory (woody weed). Plant Prod. Q..

[7] Witt A., Luke Q. (2017). Guide to the Naturalized and Invasive Plants of Eastern Africa.

[8] Plants of the World. Royal Botanical Gardens-Kew, *Mimosa pigra* L.. https://powo.science.kew.org/taxon/urn:lsid:ipni.org:names:313003-2.

[9] Miller I.L., Wilson C.G., Finlayson C.M. (1995). Weed threats to Northern Territory wetlands. Wetland Research in the Wet-Dry Tropics of Australia.

[10] Crop Protection Compendium, *Mimosa pigra* L.. http://www.mikepalmer.co.uk/woodyplantecology/docs/CPC-Mimosa_pigra.pdf.

[11] Marko M. (1999). Controlling invasion of the exotic shrub (*Mimosa pigra*) in tropical Australian wetlands. Restor. Reclam. Rev..

[12] Lonsdale W.M. (1993). Rates of spread of an invading species—*Mimosa pigra* in northern Australia. J. Ecol..

[13] Mumba M., Thompson J.R. (2005). Hydrological and ecological impacts of dams on the Kafue Flats floodplain system, southern Zambia. Phys. Chem. Earth. Part A/B/C.

[14] Shanungu G.K. (2009). Management of the invasive *Mimosa pigra* L. in Lochinvar National Park, Zambia. Biodiversity.

[15] Witt A.B., Floyd K.S., Nunda W., Beale T., Shanungu G., Kriticos D.J. (2020). *Mimosa pigra* in eastern and southern Africa: Distribution and socio-ecological impacts. Austral Ecol..

[16] Marambe B., Amarasinghe L., Silva K., Gamage G., Dissanayake S., Seneviratne A., Julien M., Flanagan G., Heard T., Hennecke B., Paynter Q., Wilson C. (2004). Distribution, biology and management of *Mimosa pigra* in Sri Lanka. Research and Management of Mimosa pigra.

[17] Pitt J.L., Miller I.L. (1988). A review of survey techniques for the detection of weeds with particular reference to *Mimosa pigra* L. Australia and Thailand. Plant Prod. Q..

[18] Rijal S., Cochard R. (2016). Invasion of Mimosa pigra on the cultivated Mekong River floodplains near Kratie, Cambodia: Farmers’ coping strategies, perceptions, and outlooks. Reg. Environ. Chang..

[19] Pramual P., Khumkratok S., Wongpakam K. (2011). Population genetics of invasive weed *Mimosa pigra* L. (Mimosoideae) in Thailand. Pak. J. Bot..

[20] Son N.H., Lam P.V., Cam N.V., Thanh D.V.T., Dung N.V., Khanh L.D., Forno I.W., Julien M., Flanagan G., Heard T., Hennecke B., Paynter Q., Wilson C. (2004). Preliminary studies on control of *Mimosa pigra* in Vietnam. Research and Management of Mimosa pigra.

[21] Mansor A., Crawley M.J. (2011). Current status of *Mimosa pigra* L. infestation in Peninsular Malaysia. Trop. Life Sci. Res..

[22] Braithwaite R.W., Lonsdale W.M., Estbergs J.A. (1989). Alien vegetation and native biota in tropical Australia: The impact of *Mimosa pigra*. Biol. Conserv..

[23] Welgama A., Florentine S., Roberts J.A. (2022). Global review of the woody invasive alien species *Mimosa pigra* (giant sensitive plant): Its biology and management implications. Plants.

[24] Heard T.A., Paynter Q., Muniappan R., Reddy G.V.P., Raman A. (2009). Mimosa pigra (Leguminosae). Biological Control of Tropical Weeds Using Arthropods.

[25] Walden D., van Dam R., Finlayson C., Storrs M., Lowry J., Kriticos D., Julien M., Flanagan G., Heard T., Hennecke B., Paynter Q., Wilson C. (2004). A risk assessment of the tropical wetland weed *Mimosa pigra* in northern Australia. Research and Management of Mimosa pigra.

[26] Haq R.U., Eiam-Ampai K., Ngoprasert D., Sasaki N., Shrestha R.P. (2018). Changing landscapes and declining populations of resident waterbirds: A 12-year study in Bung Boraphet Wetland, Thailand. Tropic. Conserv. Sci..

[27] Thamasara S. (1983). *Mimosa pigra* management in irrigation systems in Thailand. Proceedings of the International Symposium on Mimosa pigra Management.

[28] Chamroeun K., Seang P.T., Sophal H., Hout S.S., Vuthy H., McKenney B. (2002). An investigation of the impacts of *Mimosa pigra* on rice and fishery productivity in Kandal Province, Cambodia. Economy and Environment: Case Studies in Cambodia, Singapore.

[29] Mohamed M.S., Seman I.A. (2012). Occurrence of common weeds in immature plantings of oil palm plantations in Malaysia. Planter.

[30] Vitelli J.S., Madigan B.A., Worsley K.J. (2006). *Mimosa pigra* in Queensland. Proceedings of the 15th Australian Weeds Conference.

[31] Lonsdale W.M., Miller I.L., Forno I.W., Groves R.H., Shepherd R.C.H., Richardson R.G. (1995). Mimosa pigra L.. The Biology of Australian Weeds.

[32] Lonsdale W.M., Harley K.L.S. (1992). The biology of *Mimosa pigra*. A Guide to the Management of Mimosa pigra.

[33] Lonsdale W., Braithwaite R.W. (1988). The shrub that conquered the bush. New Sci..

[34] Creager R.A. (1992). Seed germination, physical and chemical control of catclaw mimosa (*Mimosa pigra* var. *pigra*). Weed Technol..

[35] van Klinken R.D., Goulier J.B. (2013). Habitat-specific seed dormancy-release mechanisms in four legume species. Seed Sci. Res..

[36] Lukitsch B., Elliott L. (2012). *Mimosa pigra* seed bank remains significant 10 years after stand removal: Further investigation on a floodplain in northern Australia. Proceedings of the 18th Australasian Weeds Conference.

[37] Marambe B., Amarasinghe L., Dissanayake S., Brundu G., Brock J., Camarda I., Child L., Wade M. (2001). Growth and development of *Mimosa pigra*: An alien invasive plant in Sri Lanka. Plant Invasions: Species Ecology and Ecosystem Manageme.

[38] Facts Sheet: Restricted Invasive Plant: Mimosa pigra: Queensland Department of Agriculture and Fisheries. https://www.daf.qld.gov.au/__data/assets/pdf_file/0007/65149/mimosa-pigra.pdf.

[39] Presnell K. (2004). The potential use of mimosa as fuel for power generation. Proceedings of the 3rd International Symposium on the Management of of Mimosa pigra.

[40] Paynter Q., Flanagan G.J. (2004). Integrating herbicide and mechanical control treatments with fire and biological control to manage an invasive wetland shrub, *Mimosa pigra*. J. Appl. Ecol..

[41] Lonsdale W.M., Miller I.L. (1993). Fire as a management tool for a tropical woody weed: *Mimosa pigra* in northern Australia. J. Environ. Manag..

[42] Thompson J.D., McNeilly T., Gray A.J. (1991). Population variation in *Spartina anglica* C.E. Hubbard. I. Evidence from a common garden experiment. New Phytol..

[43] Mack R.M. (1996). Predicting the identity and fate of plant invaders: Emergent and emerging approaches. Biol. Conserv..

[44] Chengxu W., Mingxing Z., Xuhui C., Bo Q. (2011). Review on allelopathy of exotic invasive plants. Procedia. Engin..

[45] Warren R.J., Matt Candeias M., Labatore A., Olejniczak M., Yang L. (2019). Multiple mechanisms in woodland plant species invasion. J. Plant Ecol..

[46] NurZhafarina A., Asyraf M. (2017). Effects of biotic and abiotic environmental stimuli on the morphology and biomass allocation of *Mimosa pigra* L.. Sains Malays.

[47] Harley K.L.S., Gillett J.D., Winder J., Forno I.W., Segura R., Miranda H., Kassulke R.C. (1995). Natural enemies of *Mimosa pigra* and *M. berlandieri* (Mimosaceae) and prospects for biological control of *M. pigra*. Environ. Entomol..

[48] Heard T.A., Pettit W. (2005). Review and analysis of the surveys for natural enemies of *Mimosa pigra*: What does it tell us about surveys for broadly distributed hosts?. Biol. Control..

[49] Heard T.A., Segura R., Julien M., Flanagan G., Heard T., Hennecke B., Paynter Q., Wilson C. (2004). Agents for biological control of *Mimosa pigra* in Australia: Review and future prospects. Research and Management of Mimosa pigra.

[50] Paynter Q., Julien M., Flanagan G., Heard T., Hennecke B., Paynter Q., Wilson C. (2004). Evaluating *Mimosa pigra* biological control in Australia. Research and Management of Mimosa pigra.

[51] Evans H.C., Carrión G., Guzman G. (1993). A new species of *Sphaerulina* and its *Phloeospora* anamorph, with potential for biological control of *Mimosa pigra*. Mycol. Res..

[52] Evans H.C., Carrión G., Ruiz-Belin F. (1995). Mycobiota of the giant sensitive plant, *Mimosa pigra* sensu lato in the Neotropics. Mycol. Res..

[53] Flanagan G.J., Wilson C.G., Gillett J.D. (1990). The abundance of native insects on the introduced weed *Mimosa pigra* in Northern Australia. J. Trop. Ecol..

[54] Moulin L., Munive A., Dreyfus B., Boivin-Masson C. (2001). Nodulation of legumes by members of the b-subclass of proteobacteria. Nature.

[55] Vandamme P., Goris J., Chen W.M., de Vos P., Willems A. (2002). *Burkholderia tuberum* sp. nov. and *Burkholderia phymatum* sp. nov. nodulate the roots of tropical legumes. Syst. Appl. Microbiol..

[56] Barrett C.F., Parker M.A. (2006). Coexistence of *Burkholderia*, *Cupriavidus* and *Rhizobium* sp. nodule bacteria on two *Mimosa* species in Costa Rica. Appl. Environ. Microbiol..

[57] Chen W.M., James E.K., Coenye T., Chou J.H., Barrios E., de Faria S.M., Ellio N., Sheu S.Y., Sprent J.I., Vandamme P. (2006). *Burkholderia mimosarum* sp. nov., isolated from root nodules of *Mimosa* spp. from Taiwan and South America. Int. J. Syst. Evol. Microbiol..

[58] Elliott G.N., Chou J.H., Chen W.M., Bloemberg G.V., Bontemps C., Martínez-Romero E., Velázquez E., Young J.P.W., Sprent J.I., James E.K. (2009). *Burkholderia* spp. are the most competitive symbionts of Mimosa, particularly under N-limited conditions. Environ. Microbiol..

[59] Chen W.M., James E.K., Chou J.H., Sheu S.Y., Yang S.Z., Sprent J.I. (2005). β-Rhizobia from *Mimosa pigra*, a newly discovered invasive plant in Taiwan. New Pytol..

[60] Tsyganova A.V., Brewin N.J., Tsyganov V.E. (2021). Structure and development of the legume-rhizobial symbiotic interface in infection threads. Cells.

[61] Mathesius U. (2022). Are legumes different? Origins and consequences of evolving nitrogen fixing symbioses. J. Plant Physiol..

[62] de Faria S.M., de Lima H.C. (1998). Additional studies of the nodulation status of legume species in Brazil. Plant Soil.

[63] Saur E., Carcelle S., Guezennec S., Rousteau A. (2000). Nodulation of legume species in wetlands of Guadeloupe (Lesser Antilles). Wetlands.

[64] James E.K., de Fatima Loureiro M., Pott A., Pott V.J., Martins C.M., Franco A.A., Sprent J.I. (2001). Flooding-tolerant legume symbioses from the Brazilian Pantanal. New Phytol..

[65] Chen W.M., de Faria S.M., Straliotto R., Pitard R.M., Simões-Araùjo J.L., Chou J.H., Chou Y.J., Barrios E., Prescott A.R., Elliott G.N. (2005). Proof that Burkholderia forms effective symbioses with legumes: A study of novel *Mimosa*-nodulating strains from South America. Appl. Environ. Microbiol..

[66] Willems A., Tian R., Bräu L., Goodwin L., Han J., Liolios K., Huntemann M., Pati A., Woyke T., Mavrommatis K. (2014). Genome sequence of *Burkholderia mimosarum* strain LMG 23256T, a *Mimosa pigra* microsymbiont from Anso, Taiwan. Stand. Genom. Sci..

[67] Howieson J.G., Ewing M.A., D’antuono M.F. (1988). Selection for acid tolerance in *Rhizobium meliloti*. Plant Soil..

[68] Terpolilli J.J. (2009). Why Are the Symbioses between Some Genotypes of Sinorhizobium and Medicago suboptimal for N2 Fixation?.

[69] Parker M.A., Wurtz A.K., Paynter Q. (2007). Nodule symbiosis of invasive *Mimosa pigra* in Australia and in ancestral habitats: A comparative analysis. Biol. Invasions.

[70] Rodríguez Y., Dalpé Y., Séguin S., Fernández K., Fernández F., Rivera R.A. (2011). *Glomus cubense* sp. nov., an arbuscular mycorrhizal fungus from Cuba. Mycotaxon.

[71] Santillán-Manjarrez J., Solís-Hernández A.P., Castilla-Hernández P., Maldonado-Mendoza I.E., Vela-Correa G., Chimal-Hernández A., Hernández-Díaz C., Signoret-Poillon M., Diederik van Tuinen D., Rivera-Becerril F. (2019). Exploring plant root-fungal interactions in a neotropical freshwater wetland. Bot. Sci..

[72] Davison J., Öpik M., Daniell T.J., Moora M., Zobel M. (2011). Arbuscular mycorrhizal fungal communities in plant roots are not random assemblages. FEMS Microbiol. Ecol..

[73] Smith S.E., Read D.J. (2008). Mycorrhizal Symbiosis.

[74] Diagne N., Ngom M., Djighaly P.I., Fall D., Hocher V., Svistoonoff S. (2020). Roles of Arbuscular Mycorrhizal Fungi on Plant Growth and Performance: Importance in Biotic and Abiotic Stressed Regulation. Diversity.

[75] Tang H., Hassan M.U., Feng L., Nawaz M., Shah A.N., Qari S.H., Liu Y., Miao J. (2022). The critical role of arbuscular mycorrhizal fungi to improve drought tolerance and nitrogen use efficiency in crops. Front. Plant Sci..

[76] Ramírez-Viga T.K., Aguilar R., Castillo-Argüero S., Chiappa-Carrara X.L., Guadarrama P., Ramos-Zapata J. (2018). Wetland plant species improve performance when inoculated with arbuscular mycorrhizal fungi: A meta-analysis of experimental pot studies. Mycorrhiza.

[77] Callaway R.M., Aschehoug E.T. (2000). Invasive plants versus their new and old neighbors: A mechanism for exotic invasion. Science.

[78] Callaway R.M., Ridenour W.M. (2004). Novel weapons: Invasive success and the evolution of increased competitive ability. Front. Ecol. Environ..

[79] Cappuccino N., Arnason J.T. (2006). Novel chemistry of invasive exotic plants. Biol. Lett..

[80] Meiners S.J., Kong C.H., Ladwig L.M., Pisula N.L., Lang K.A. (2012). Developing an ecological context for allelopathy. Plant. Ecol..

[81] Rice E.L. (1984). Allelopathy.

[82] Bais H.P., Weir T.L., Perry L.G., Gilroy S., Vivanco J.M. (2006). The role of root exudates in rhizosphere interactions with plants and other organisms. Annu. Rev. Plant Biol..

[83] Bonanomi G., Sicurezza M.G., Caporaso S., Esposito A., Mazzoleni S. (2006). Phytotoxicity dynamics of decaying plant materials. New Phytol..

[84] Belz R.G. (2007). Allelopathy in crop/weed interactions—An update. Pest. Manag. Sci..

[85] Kato-Noguchi H. (2020). Involvement of allelopathy in the invasive potential of *Tithonia diversifolia*. Plants.

[86] Kato-Noguchi H., Kurniadie D. (2021). Allelopathy of *Lantana camara* as an invasive plant. Plants.

[87] Kato-Noguchi H. (2022). Allelopathy of knotweeds as invasive plants. Plants.

[88] Kato-Noguchi H., Kurniadie D. (2022). Allelopathy and allelochemicals of *Leucaena leucocephala* as an invasive plant species. Plants.

[89] Kato-Noguchi H. (2022). Allelopathy and allelochemicals of *Imperata cylindrica* as an invasive plant species. Plants.

[90] Kato-Noguchi H., Kato M. (2022). Allelopathy and allelochemicals of *Solidago canadensis* L. and *S. altissima* L. for their naturalization. Plants.

[91] Kato-Noguchi H., Kato M. (2023). Evolution of the secondary metabolites in invasive plant species *Chromolaena odorata* for the defense and allelopathic functions. Plants.

[92] Koodkaew I., Rottasa R. (2017). Allelopathic effects of giant sensitive plant (*Mimosa pigra*) leaf powder on germination and growth of popping pod and purslane. Int. J. Agric. Biol..

[93] Oso O.A., Ajayi B., Okanume O.E. (2022). Descriptive anatomy of invasive weed, *Ruellia tuberosa* Linn. Asian J. Res. Bot..

[94] Prasangani W.D., Sinhalage I.D., Karunathilake A.A., Madusinghe M. Phytotoxicity Effect of three invasive plant species: *Mimosa pigra*, *Parthenium hysierophorus*, *Ulex europaeus*. Proceedings of the Research Symposium of Uva Wellassa University.

[95] Araújo M.S., Santos S.P., Barros-Filho B.A., Lima M.M.O., Leite A.S. (2020). Toxicogenetic potential of *Mimosa pigra* (Fabaceae) infusion in *Allium cepa* meristematic cells. Genet. Mol. Res..

[96] Koodkaew I., Wannathong R. (2018). Effects of *Mimosa pigra* L. leaf extract on growth behavior of *Ruellia tuberosa* L. and *Echinochloa crus-galli* (L.) P. Beauv. Asia-Pac. J. Sci. Technol..

[97] Koodkaew I., Senaphan C., Sengseang N., Suwanwong S. (2018). Characterization of phytochemical profile and phytotoxic activity of *Mimosa pigra* L.. Agric. Nat. Resour..

[98] Lonsdale W.M. (1988). Litterfall in an Australian population of *Mimosa pigra*, an invasive tropical shrub. J. Trop. Ecol..

[99] Renz J. (1936). Mimosine. Physiol. Chem..

[100] Brewbaker J.L., Hylin J.W. (1965). Variations in mimosine content among *Leucaena* species and related mimosaceae. Crop Sci..

[101] Smith I.K., Fowden L.A. (1996). Study of mimosine toxicity in plants. J. Exp. Bot..

[102] Soedarjo M., Borthakur D. (1996). Mimosine produced by the tree-legume Leucaena provides growth advantages to some *Rhizobium* strains that utilize it as a source of carbon and nitrogen. Plant Soil.

[103] Nguyen B.C., Tawata S. (2016). The chemistry and biological activities of mimosine: A review. Phytother. Res..

[104] Rakotomalala G., Agard C., Tonnerre P., Tesse A., Derbré S., Michalet S., Hamzaoui J., Rio M., Cario-Toumaniantz C., Richomme P. (2013). Extract from *Mimosa pigra* attenuates chronic experimental pulmonary hypertension. J. Ethnopharm..

[105] De Morais C.B., Scopel M., Pedrazza G.P.R., da Silva F.K., Dalla Lana D.F., Tonello M.L., Miotto S.T.S., Machado M.M., De Oliveira L.F.S., Fuentefria A.M. (2017). Anti-dermatophyte activity of Leguminosae plants from Southern Brazil with emphasis on *Mimosa pigra* (Leguminosae). J. Mycol. Med..

[106] Rizwan K., Majeed I., Bilal M., Rasheed T., Shakeel A., Iqbal S. (2022). Phytochemistry and diverse pharmacology of Genus *Mimosa*: A review. Biomolecules.

[107] Okonkwo C.J., Njoku O.U., Okonkwo T.J., Afieroho O.E., Proksch P. (2016). Two new acylated flavonol glycosides from *Mimosa pigra* L. leaves sub-family Mimosoideae. Future J. Pharm. Sci..

[108] Hawwal M.F., Ali Z., Fantoukh O.I., Chittiboyina A.G., Khan I.A. (2021). Phytochemical investigation of Mimosa pigra leaves, a sensitive species. Biochem. Syst. Ecol..

[109] Englert J., Weniger B., Lobstein A., Anton R., Krempp E., Guillaume D., Leroy Y. (1995). Triterpenoid saponins from *Mimosa pigra*. J. Nat. Prod..

[110] Nguyen L.N.P., Huu D.M.N., Dang H.P., Huynh N.V., Dang P.H. (2021). A new furanochromone from the leaves of *Mimosa pigra*. Nat. Prod. Res..

[111] Treutter D. (2006). Significance of flavonoids in plant resistance: A review. Environ. Chem. Lett..

[112] Weston L.A., Mathesius U. (2013). Flavonoids: Their structure, biosynthesis and role in the rhizosphere, including allelopathy. J. Chem. Ecol..

[113] Mierziak J., Kostyn K., Kulma A. (2014). Flavonoids as important molecules of plant interactions with the environment. Molecules.

[114] Santonja M., Le Rouzic B., Thiébaut G. (2018). Seasonal dependence and functional implications of macrophyte-phytoplankton allelopathic interactions. Freshw. Biol..

[115] Thouvenot L., Haury J., Thiébaut G. (2013). A success story: Water primroses, aquatic plant pests. Aquatic Conservation Mar. Freshw. Ecosyst..

[116] Okada S., Iwasaki A., Kataoka I., Suenaga K., Kato-Noguchi H. (2018). Isolation and identification of a phytotoxic substance in kiwifruit leaves. Acta Hortic..

[117] Parvez M.M.K., Yokotani T., Fujii Y., Konishi T., Iwashina T. (2004). Effects of quercetin and its seven derivatives on the growth of *Arabidopsis thaliana* and *Neurospora crassa*. Biochem. Syst. Ecol..

[118] Moody M.E., Mack R.N. (1988). Controlling the spread of plant invasions: The importance of nascent foci. J. Appl. Ecol..

[119] Lake E.C., Minteer C.R. (2018). A review of the integration of classical biological control with other techniques to manage invasive weeds in natural areas and rangelands. BioControl.

[120] Siriworakul M., Schultz G.C., Harley K.L.S. (1992). Physical and Mechanical Control of Mimosa pigra. A Guide to the Management of Mimosa pigra.

[121] DiTomaso J.M., Kyser G.B., Miller J.R., Garcia S., Smith R.F., Nader G., Connor J.M., Orloff S.B. (2006). Integrating prescribed burning and clopyralid for the management of yellow starthistle (*Centaurea solstitialis*). Weed Sci..

[122] Drus G.M., Dudley T.L., Brooks M.L., Matchett J. (2013). The effect of leaf beetle herbivory on the fire behaviour of tamarisk (*Tamarix ramosissima* Lebed.). Int. J. Wildland Fire.

[123] Miller I.L., Siriworakul M., Harley K.L.S. (1992). Herbicide research and recommendations for control of *Mimosa pigra*. A Guide to the Management of Mimosa pigra.

[124] (2013). Department of Land Resource Management. Weed Management Plan for Mimosa (*Mimosa pigra*). https://denr.nt.gov.au/__data/assets/pdf_file/0016/400372/Final-Weed-Management-Plan-for-Mimosa-Dec-2013.pdf.

[125] Lane A.M., Williams R.J., Muller W.J., Lonsdale W.M. (1997). The effects of the herbicide tebuthiuron on seedlings of *Mimosa pigra* and native floodplain. Austral. J. Ecol..

[126] Ostermeyer N., Grace B.S. (2007). Establishment, distribution and abundance of *Mimosa pigra* biological control agents in northern Australia: Implications for biological control. BioControl.

[127] Burrows N., Lukitsch B. (2012). Biological control agents are observed on *Mimosa pigra* six and 12 years after their release in the Northern Territory, Australia. Proceedings of the 18th Australasian Weeds Conference.

[128] Paynter Q. (2005). Evaluating the impact of a biological control agent *Carmenta mimosa* on the woody wetland weed *Mimosa pigra* in Australia. J. Appl. Ecol..

[129] Wilson C.G., Flanagan G.J. (1992). Establishment and spread of *Neurostrota gunniella* on *Mimosa pigra* in the Northern Territory. Proceedings of the 9th Australasian Weeds Conference.

[130] Davis D.R., Kassulke R.C., Harley K.L.S., Gillett J.D. (1991). Systematic, morphology, biology, and host specificity of *Neurostrota gunniella* (Busk) (Lepidoptera: Gracillariidae), an agent for the biological control of *Mimosa pigra* L.. Proc. Entomol. Soc. Wash..

[131] Paynter Q., Hennecke B. (2001). Competition between two biological control agents, *Neurostrota gunniella* and *Phloeospora mimosae-pigrae*, and their impact on the invasive tropical shrub *Mimosa pigra*. Biocontrol Sci. Technol..

[132] Paynter Q. (2006). Evaluating the impact of biological control against *Mimosa pigra* in Australia: Comparing litterfall before and after the introduction of biological control agents. Biol. Control..

[133] Heard T.A., Elliott L.P., Anderson B., White L., Burrows N., Mira A., Zonneveld R., Fichera G., Chan R., Segura R. (2010). Biology, host specificity, release and establishment of *Macaria pallidata* and *Leuciris fimbriaria* (Lepidoptera: Geometridae), biological control agents of the weed *Mimosa pigra*. Biol. Control..

[134] Heard T.A., Forno W.I. (1996). Host selection and host range of the flower-feeding weevil, *Coelocephalapion pigrae*, a potential biological control agent of *Mimosa pigra*. Biol. Control..

[135] Heard T.A., Paynter Q., Chan R., Mira A. (2005). *Malacorhinus irregularis* for biological control of *Mimosa pigra*: Host specificity, life cycle and establishment in Australia. Biol. Control..

[136] Burrows N.J., Lukitsch B.V., Liberato J.R. (2012). Rediscovery of the rust *Diabole cubensis*, released as a classical biological control agent against the invasive weed *Mimosa pigra* in Australia. Australas. Plant Dis. Notes.

[137] Suasa-ard W., Sommartya P., Jaitui S., Julien M., Flanagan G., Heard T., Hennecke B., Paynter Q., Wilson C. (2004). Evaluation of seed-feeding bruchids, *Acanthoscelides* species, as biological control agents for *Mimosa pigra* in Thailand. Research and Management of Mimosa pigra.

[138] Buckley Y., Rees M., Paynter Q., Lonsdale M. (2004). Modelling integrated weed management of an invasive shrub in tropical Australia, *Mimosa pigra*. J. Appl. Ecol..

[139] van Driesche R. (2012). The role of biological control in wildlands. BioControl.

[140] Mosley J.C., Frost R.A., Roeder B.L., Mosley T.K., Marks G. (2016). Combined herbivory by targeted sheep grazing and biological control insects to suppress spotted knapweed (*Centaurea stoebe*). Invasive Plant Sci. Manag..

[141] Hatcher P., Melander B. (2003). Combining physical, cultural and biological methods: Prospects for integrated non-chemical weed management strategies. Weed Res..

[142] Lym R.G. (2005). Integration of biological control agents with other weed management technologies: Successes from the leafy spurge (*Euphorbia esula*) IPM program. Biol. Control.

